# Stability study of flame structure using photodiodes in surface flame burners

**DOI:** 10.1038/s41598-024-55545-0

**Published:** 2024-03-06

**Authors:** Parmis Sadat Jazayeri, Mohammad Zabetian Targhi, Mohammad Reza Karafi

**Affiliations:** Faculty of Mechanical Engineering, Tarbiat Modares University, Tehran, Iran

**Keywords:** Stability, Pollutant, Optical detector, Radiation spectroscopy, Perforated burner, Energy infrastructure, Mechanical engineering

## Abstract

Radiation spectroscopy can be effective in identifying pollutants and species. By examining a photodiode, the frequency peaks obtained from the analysis, and the range in which the cell flame is converted to a surface flame, we obtained the value ratio of the cell flame to the surface flame in the range of 0.7–0.74 in different powers. The frequency peak in this range decreases from the maximum value of 9.9823 Hz to its minimum value of 9.058 Hz in different powers. By analyzing the temperature compared to the frequency peak, we found that in the range of equivalence ratio 0.7–0.75, the frequency peak decreases from 9.5 to 9.9 Hz to 8.7–9 Hz. The temperature has an increasing behavior, and in the equivalence ratio, the temperature is in the range of 1400–1500 °C, i.e., at its maximum value. We observed the cell formation process and its conversion to surface flame by flame detection. The flame height in the cellular and superficial regions is in the range of 0.65–0.85, which is the minimum flame height of 3–10 mm, and *NO* and *CO* were examined in the ratio of different equations and compared with temperature. In the ratio equivalence ratio of 0.77–0.81 in the temperature range of 1500 °C (maximum), the value of *NO* is about 16 ppm (maximum), and the value of *CO* is about 2 ppm (minimum). That is, when the temperature is at its maximum, it becomes *CO* minimum and *NO* maximum. This can be used for different applications such as similar and industrial burners.

## Introduction

Environmental problems and energy crises have increased demand for less polluting and more efficient domestic and industrial combustion systems. To achieve this goal, new technologies tend to premix and dilute combustion. Among these technologies, surface flame burners are widely used in domestic and industrial combustion.

In recent years, porous burners in home use applications have attracted much attention for putting experiments together with simulations that concern emission and temperature distribution^1^. It is, therefore, paving the way for using the tool of computations and experiments to achieve the merits of porous combustion for porous cooking burners^2,3^. In addition to emissions and temperature, alternative fuels for domestic use are attractive and can more effectively respond to future demand^4^.

Non-contact methods (including radiation measurement by photodetector) can measure more accurately and efficiently even for remote points. Some aspects of research innovation are:

1. Detection of flame pollutants using a photodetector
2. Using flame radiation to detect combustion species
3. Measurement of flame temperature and composition using natural flame radiation
4. Using optical detectors to measure flame radiation

One of the most essential features of these burners is the creation of small flames. Due to the creation of these small flames, heat transfer is uniform, and as a result, the amount of NOx and carbon dioxide is reduced. The flame becomes more stable by creating small flames in these burners and transferring their heat to the burner plate, which has more conduction. In addition, measurements show that radiation has the largest share in the heat transfer of these burners^5^. Also, the fresh mixture is preheated in contact with the burner plate, which has more significant heat transfer, increasing efficiency. By optimizing the flame stability parameters (flame adhering to the surface without rising and without extinguishing), the amount of NOx and carbon monoxide in the surface flame of a cylindrical flame with natural gas fuel determines the optimal equivalence range between 0.7 and 0.75. The state forms a blue flame^6^. Surface flame burners are widely used for basic combustion studies.

Soltanian et al.^1^ the flame radiation spectroscopy method was investigated in order to detect combustion species in a premixed perforated burner. The spectrometer plots the intensity versus wavelength by computer. A black fireproof sheet is used to reduce the background light. The exposure time was considered to be 10 ms to increase the signal to noise.

Surface flame burners are a class of burners that use surface flame interaction to improve burner performance. Combustion researchers today focus on developing new measurement and detection methods to achieve higher efficiencies and reduce fuel consumption and combustion pollutants that meet the new challenges of the combustion industry, including increasing efficiencies, reliability, and flexibility (use of new fuels and reduction of environmental impacts). Along with developing new burner designs and concepts, optimizing the flame as the combustion core is vital. Common tools are used to measure variables such as the inflow rate and specific species concentrations in exhaust gases (such as *NO*x, *CO*, *O*2, and S*O*2). However, these instruments provide a minimal description of the process performed in combustion chambers, plus only at the end of the process. Combustion can be seen in these measurements, and malfunctions during combustion are undetected.

Accurate diagnoses require much more information than flame characteristics, so control tools are constantly needed to monitor whether the system is operating as expected. One method of examining a burner is to analyze the light emitted by the flame. Light emitted from the flame can be detected spontaneously or by an external energy source stimulation. One of the methods for detecting combustion parameters is non-interference. Flame radiation is measured using fiber optic, photodiode, or camera methods. The detection of flame instability in premixed flames by time series analysis and using frequency analysis by the photodiode is done so that analyzing a set of typical oscillation frequencies (peak frequency) can be ratios of the same recognized currency in which the flame is unstable. The instability of premixed flames is observed in experiments as cellular flames^7^. The intrinsic instability of premixed flames mainly includes the hydrodynamic effects of thermal expansion between the flame fronts and the perturbation-temperature effects of mass penetration and overheat penetration (Lewis number less than one)^8^.

Flame instability can be detected using numerically investigated unstable two-dimensional equations of reaction currents based on the Navier–Stokes equation, assuming a constant density based on the perturbation-temperature model, laboratory methods, or time series analysis methods. We can estimate volatility levels by time series and frequency analysis. Time series analysis examines flame instability differently^8^.

The story of our work comes from studies by Kaewpradap et al., which started in 2007. The effects of heat dissipation on the turbulent behavior of a cellular premix flame due to the inherent instability of these flames were numerically studied using two-dimensional unstable equations of reaction flows based on the Navier–Stokes equations^9^. He states that this inherent instability is due to the Lewis number being less than one. In other words, the mass penetration exceeds the heat penetration and causes heat loss. He performed a time series analysis of slow flame velocity fluctuations and observed a peak frequency in the amplitude diagram in terms of frequency. This peak frequency is the normal frequency of flame velocity fluctuations^9^.

Kadowaki et al. continued the work of Kaupradap and Gotoda in 2008, this time examining the time series of light intensity received from a dilute premix flame. The light received from the flame is the same light emitted by the flame radicals; therefore, it dramatically affects flame dynamics and, subsequently, the inherent instability of dilute premixed flames. By examining the intensity of light received from the flame in relation to time, he devised a method by which the flame's instability can be estimated. Kadovaki showed that the lower the equivalence ratio, the more premixed the flame, which leads to cellularization and instability. The fluctuations of the received light intensity with time in the equivalence ratio are 0.66, more severe than the equivalence ratio 1. He attributes this to the dilute flame's inherent instability and the heat loss effects due to the mass infiltration over the heat infiltration. That is why there are much more intense fluctuations in the equivalence ratio of 0.66 and the flow rate of 40 L/min; increasing the flow rate increases the effect of instability due to Lewis’s number being less than one. By performing FFT on the time series, he showed that the peak frequency is also lower at low equivalence ratios. This peak frequency is the same as the general frequency of premixed flames, mainly due to the shear current between hot burned gases and atmospheric gases^7^.

In 2010, Kaewpradap et al. continued their work (2007) on oxygen flames, investigating the effect of homogeneity and CO2 ratio on flame stability. By changing the stoichiometry and diluting the mixture, the flame moved towards the cellular flame. The reason for the inherent instability of the flame is the diluted equivalence ratio^10^.

In 2019, Kaewpradap et al. conducted an experimental study on two types of natural gas in Thailand, increasing flame stability in line with previous works and using a McKenna flat burner and a porous ceramic burner. Eastern natural gas combustion with the McKenna flat burner has a more stable flame with a smaller cell size and a wider cell flame ignition limit than Western natural gas^11^. The porous ceramic burner should have a more stable flame without flashback, which should be increased to the equivalence ratio of 0.75^11^.

In 2021, Kaupradap et al. experimentally investigated the properties of hydrogen-propane-butane-air mixture flames in continuation of their previous research on a flat burner. The cells’ width, the peak frequency of direct imaging, and the obtained light emission intensity time series were used to describe the fluctuation of mixed flames. The obtained results show the premixed flame’s unsteady characteristics for controlling lean premixed flames^12^.

In this research, the use of photodiodes with higher wavelengths and the use of various other forms of burners can be seen.

The most critical research gaps in flame processing were easy access to the flame, failure to investigate flame behavior using photodiodes, and examination of burner contaminants using photodiodes. In the first two cases, with the help of a photodiode, we had easier access to the flame. We studied the behavior of the flame as cellular and superficial and found out in which ratios the flame's value is more stable, and in a stable range, it manifests itself as a surface flame. In the latter case, however, due to our restrictions on purchases from other countries, we could not provide high-wavelength photodiodes for pollutants.

## Testbeds and laboratory equipment

**Figure 1:**
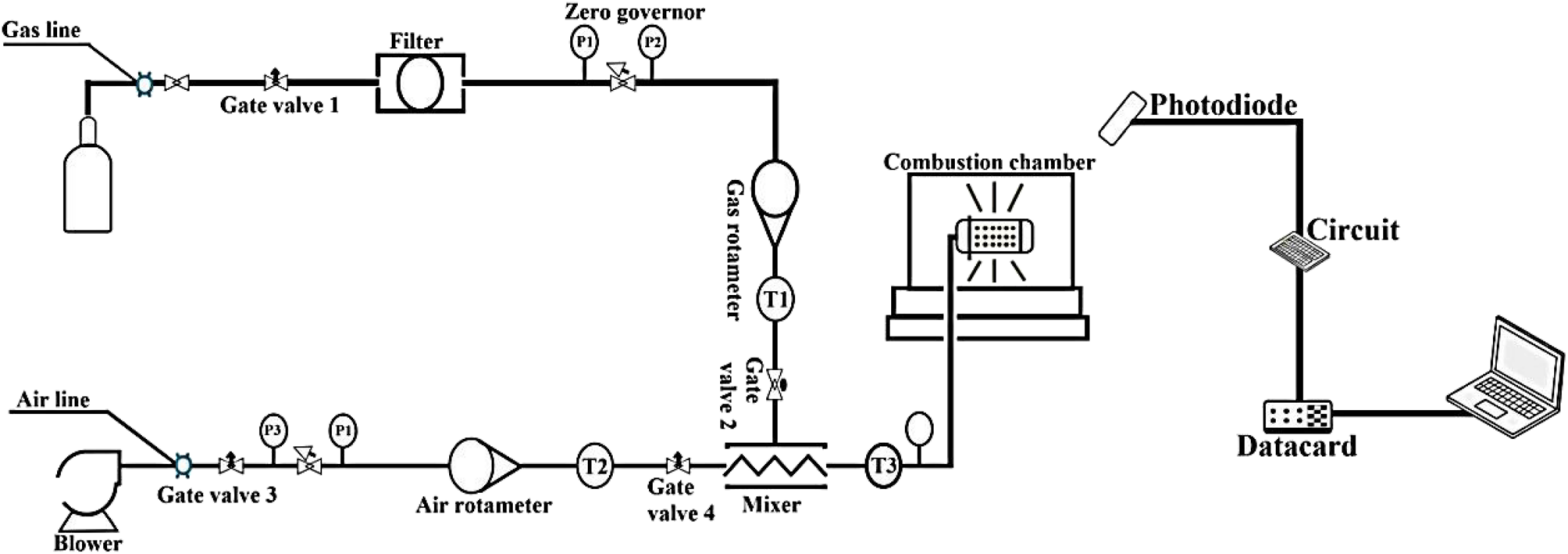
Schematic view of the arrangement of components together.

The blower used in the test of the RT-3009-1 model, made by PEM with a power of 0.75 kW and an engine speed of 2830 rpm, has been used to feed the airline with a diameter of 1 inch. After the blower, the air goes through a control valve and a regulator and enters the rotameter flowmeter with an acrylic body with an accuracy of 2% of the total scale. Finally, passing air through the fourth T control valve enters the PMG 120 mixer with all 1.5- to 5-in. dimensions. The measuring range of the rotameter is 0.5–4.4 m^3^/h. The thermometers used are of the bimetallic type, with a range of 0–60 °C and an accuracy of 1 °C (Fig. 1).

**Figure 2:**
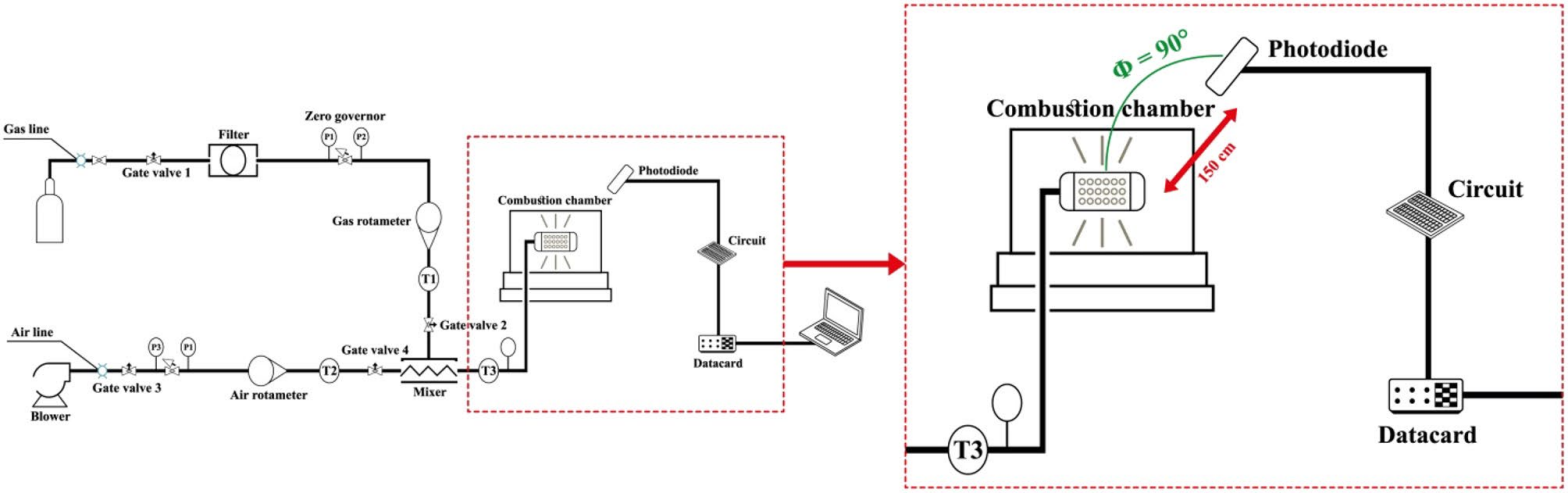
The angle between the burner and the photodetector.

To design a combustion chamber, the temperature of the flame and hot gases inside and near the inner wall are required. Due to the temperature limit of its sensor, it is necessary to use a gas analyzer to thermometer the products of combustion output^13^. Also, the intensity ratios are required to validate the method to determine the flame temperature. The S-type thermocouple was used to measure temperatures below 1600 °C and the B-type thermocouple was used to measure temperatures above 1850 °C. The flame temperature is high in some ratios of all values, so a B-type thermocouple is used. Type S thermocouples are also used to measure combustion gases inside the chamber (Fig. 2).

**Figure 3:**
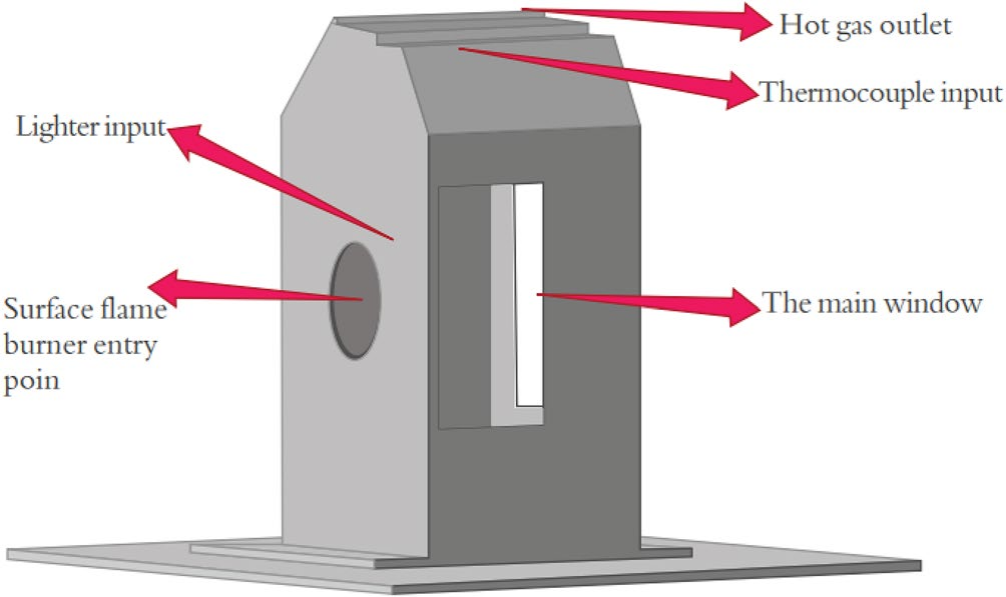
Conceptual design of the enclosure for construction.

To measure the temperature of the combustion chamber body, a gun radial thermometer (professional laser thermometer model Testo 845) is used, which is an infrared data thermometer with the ability to select optical resolution at short and long distances and with the ability to connect thermocouples with high accuracy and quality. This thermometer can also be used to obtain the burner temperature. A Testo gas analyzer, which has a tiny laboratory space and operates based on physical or physicochemical changes, is used to measure combustion products. Different species are converted into electrical signals during processes such as magnetic effects, transmission, absorption, ionization, conduction, heat, and the calibration process of gas concentrations. High-resolution cameras (25–30 frames per s) are used to capture the flame. The conceptual design of the site for this research is shown in Fig. 3.

Using a photodiode, the intensity of the received light can be obtained in terms of time. The current of the BPX 65 photodiode is converted to a voltage by a resistor. The specifications of photodiodes are presented in Table 1. This voltage is amplified 10,000 times by an amplifier and finally connected to the ADV/USB 4704-AE data collection system, and based on the 14-bit rate of the data collection system, the intensity time-series signal. The received light is stored digitally with a specific resolution. Then, using a fast Fourier transform, the light intensity is stored in terms of frequency. The photodiode board is located at a distance of 40 cm from the surface of the burner. The temporal and frequency data rates are 10 s and 10 kHz, respectively. Experiments are performed at an ambient temperature of 27 °C, an ambient pressure of 88.5 kPa, a discharge range of 1.6–1.1 cubic meters per hour, and a burner capacity of 10–20 kW. An uncertainty analysis of the experimental data was performed, and the acceptable level was calculated below the relative standard deviation of 10% of the data.

**Table 1:** Specifications of the photodiodes.

Section	Scale (cm)
Enclosure installation page	50*50
Overall dimensions of the enclosure	25*20*20
Main window	15*10
Thermocouple input	1/5
Hot gas outlet	8*20

### Amplifier circuits and signal processing

The nominal capacity of the burner used in the device is 28 kW. A range of different burner capacities and equilibrium ratios are considered to cover the different combustion regimes used for the perforated burner head. Based on this, different combustion states of the flame can be divided into four groups: turquoise flame, bright yellow flame, steady blue flame, and rising flame, which have a high-to-low ratio of equivalence^14^. To obtain different equivalence ratios at the constant power of the burner, as mentioned earlier, the airflow rate must be changed by keeping the inlet fuel flow constant. The accuracy of airline and fuel rotameters is the criterion for determining the equivalence ratio value^15^. In this way, the flow rate is placed on their main rating, and the equivalence ratio is calculated from it. This is because the equivalence ratio is reported more accurately in this case. The equivalence ratios in all experiments vary between 0.4 and 1.65. The burner's power, which is determined directly from the fuel flow, has been stabilized at values of 10.44, 11.39, 12.34, 13.28, 14.23, and 15.18 kW, and the same values have been used in all tests.

Since at high flow rates, the error rate of rotameters increases (2% of the maximum scale), gas flow is between the minimum and maximum values of 1.1 and 1.6 m^3^/h, and airflow is between a minimum of 9 cubic meters and a maximum of 35 cubic meters, restricted on all tests. The steps for changing the gas flow in each test are 0.1 cubic meters per hour, and for airflow, it is one cubic meter per hour. Among the two flammability limits, one refers to the low limit (low equivalence ratio) and the dilute flame, and the other to the high limit and the rich flame. Investigation and range of the above two limits, considering that the fully premixed chamber leads to flame extinction.

The specifications of the photodiodes used in this research are presented in Table 2.

**Table 2:** Specifications of the photodiodes.

Characteristic	Range			Unit
	BPX 65 model	PD348C (black)	BPW46 (white)	
Sensitivity range	350–1100	400–1200	430–1100	nm
Operating temperature	− 40 to 125	− 40 to 140	− 25 to 115	°C
Light-sensitive surface	1.00	1.00	1.00	mm^2^
Divergence angle	40	30	40	deg

**Figure 4:**
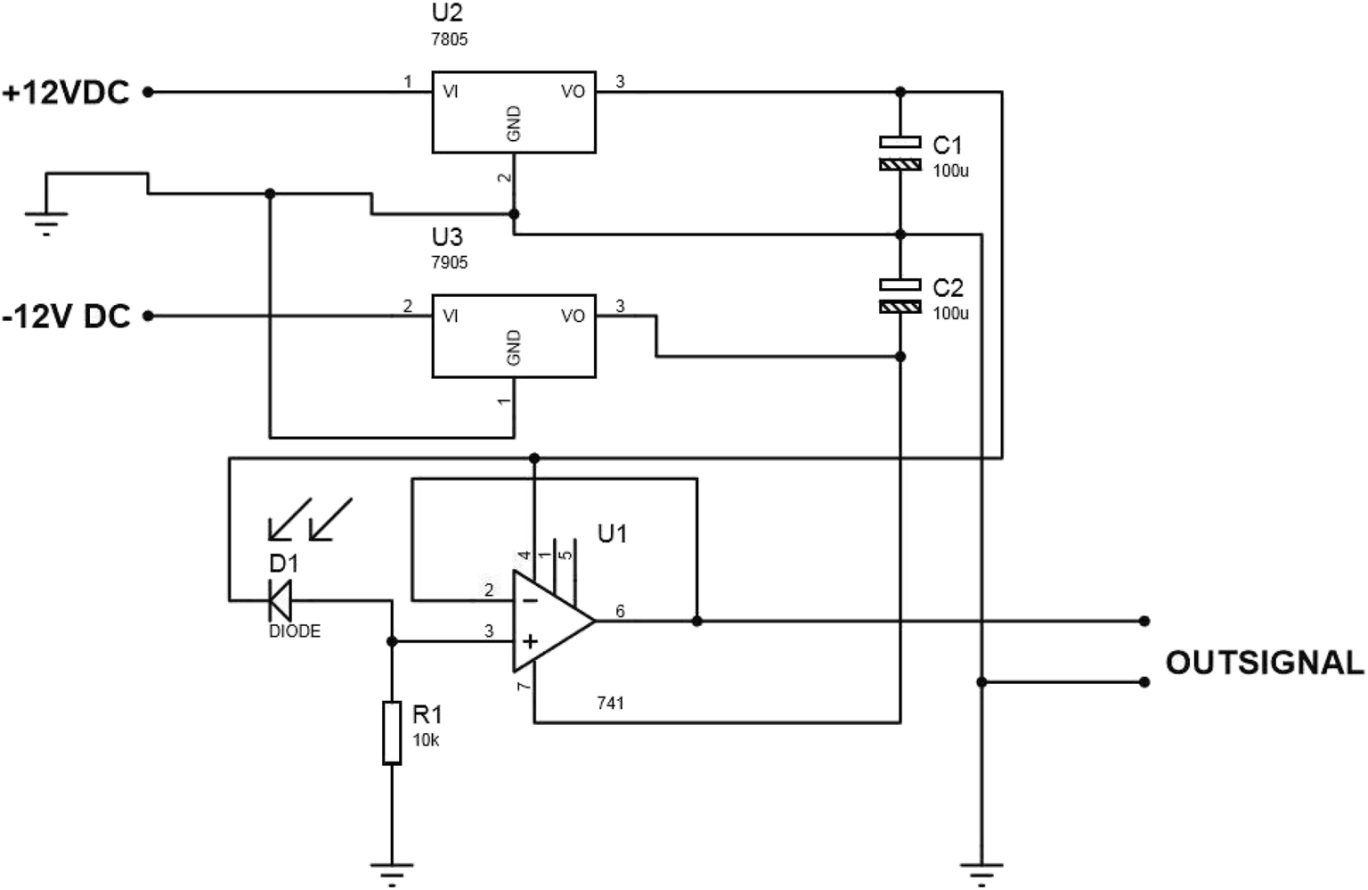
BPX65 dual-photodiode circuit.

An 8-pin op-amp module uses a photodiode, each with a particular number and a specific task. Two adapters were connected in series to facilitate power supply and easy connection to the mains and to receive positive and negative voltage. The dual-photodiode circuit used in this study is shown in Fig. 4.

In the field of optical measurements in the testbed, the calibration of the photodiode is of particular importance for reporting the true spectrum of radiation received. The spectra obtained from the photodiode are raw and must be calibrated using the reference spectrum.

A light source with a specified power was used in a dark room to calibrate the photodiode. This light source must be positioned in the same position as the torch relative to the photodiode test circuit to calibrate the photodiode in actual test conditions. Accordingly, the light source is placed at a distance of 40 cm from the photodiode test circuit, and the data collection system records the temporal distribution of light intensity, and this spectrum is considered the reference spectrum. The data collection time for each test is 10 s.

## Results and discussion

**Figure 5:**
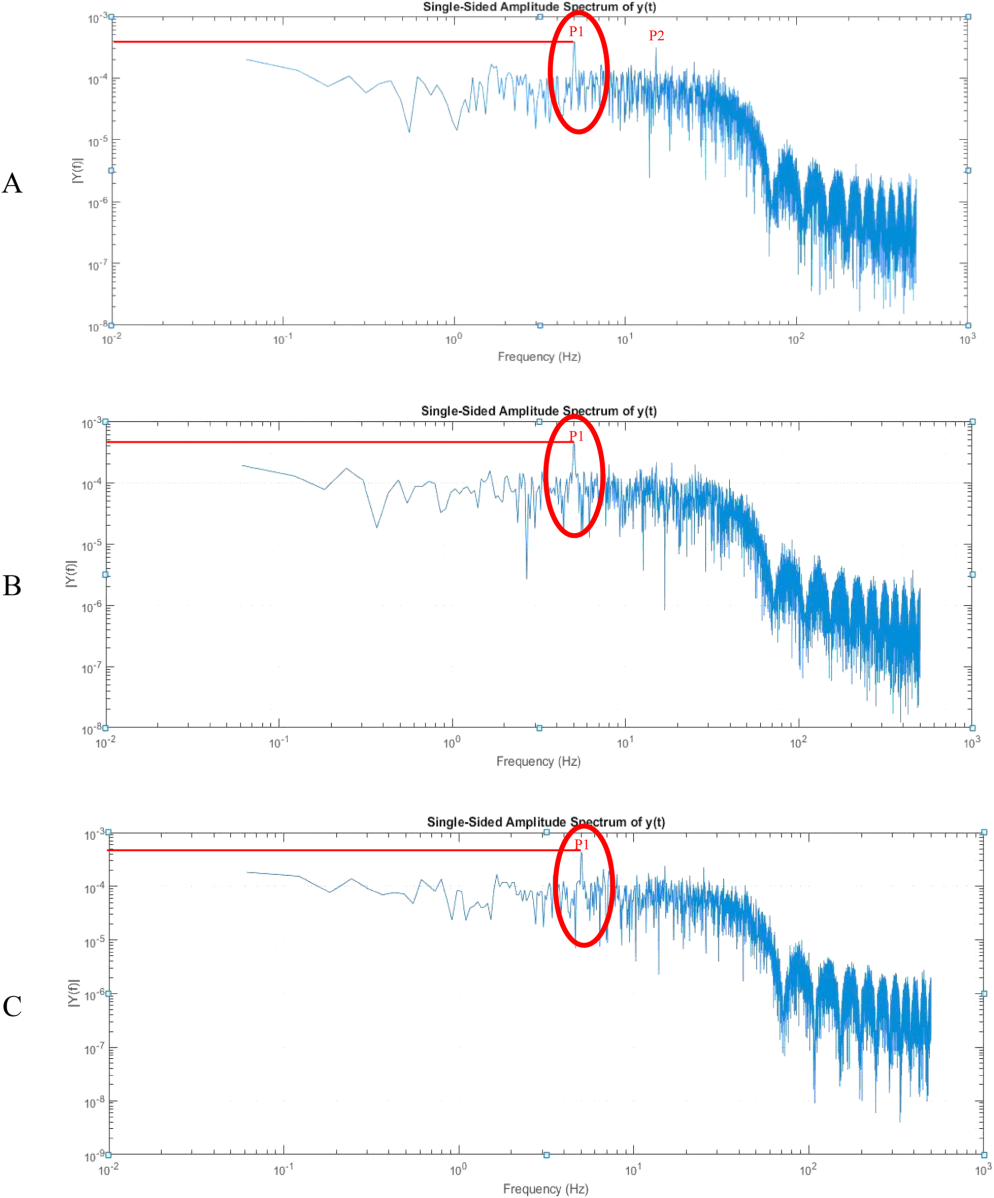
Spectral power diagram of a photodiode with an equivalence ratio of 0.79 and a heat capacity of 12.77 kW. (**A**) BP65; (**B**) PD438C; (**C**) BPW46.

After passing the transient state and reaching the steady state in the spectral density curve, a band or frequency range is considered where the range, maximum amplitude, or peak occurs for different flames with different regimes. In this curve, there is a main peak frequency and a series of harmonic peak frequencies, which indicate the deviation of the flame from the central axis and the change in the rotation frequency of the input current. The light intensity is stored in time by a data card in the computer, and the fast Fourier transform code transmits these signals in MATLAB software space from time–space to frequency-space. The basis for choosing the peak frequency is considering the absolute maximum of the frequency peaks in the frequency range. This frequency range is considered in premixed and quiet flames, from 7 to 11 Hz (Fig. 5). This range is included in similar articles, such as those by Kadovaki et al.^7^.

The detection of flame instability in premixed flames is performed by time series analysis using frequency analysis. Analyzing a set of natural oscillation frequencies (peak frequencies) makes it possible to identify equivalence ratios in which the flame is unstable. Premixed flame instability is observed in experiments as cellular flames. The oscillations’ natural frequency indicates the flame front’s rotational characteristics. Dynamic flame behavior is complex. In order to show this behavior for the premix flame, the premix flames are divided into two areas: 1-cellular flames and 2-planar flames. This taxonomy depends on the heat capacity (flow rate) and the equivalence ratio^10^. In a surface flame, as the flow rate (heat capacity) increases, so does the natural frequency of the oscillations because, as the flow rate increases, the velocity of the hot gases burned increases. In cell flames, the natural frequency of oscillations decreases with an increasing current rate (heat capacity).

In the low-frequency range, the peak frequency of the oscillation is inversely related to its amplitude. According to the concept of hertz, which is the number of oscillations per second, in fact, in the low-frequency range, stable flames, which have a greater amplitude of oscillations and a lower frequency, are considered. This is why a radiant yellow flame, which has a flame attached to the surface, is more stable.

As the thermal capacity increases, the frequency peak amplitude decreases due to the shrinkage and gradual disappearance of the yellow radiant flame due to the rising unburned mixture flow rate with increasing capacity. Therefore, the mixing speed is faster than the burning speed. As a result, the flame moves away from the burner surface, and surface flushing is eliminated, accompanied by a decrease in the peak frequency.

**Figure 6:**
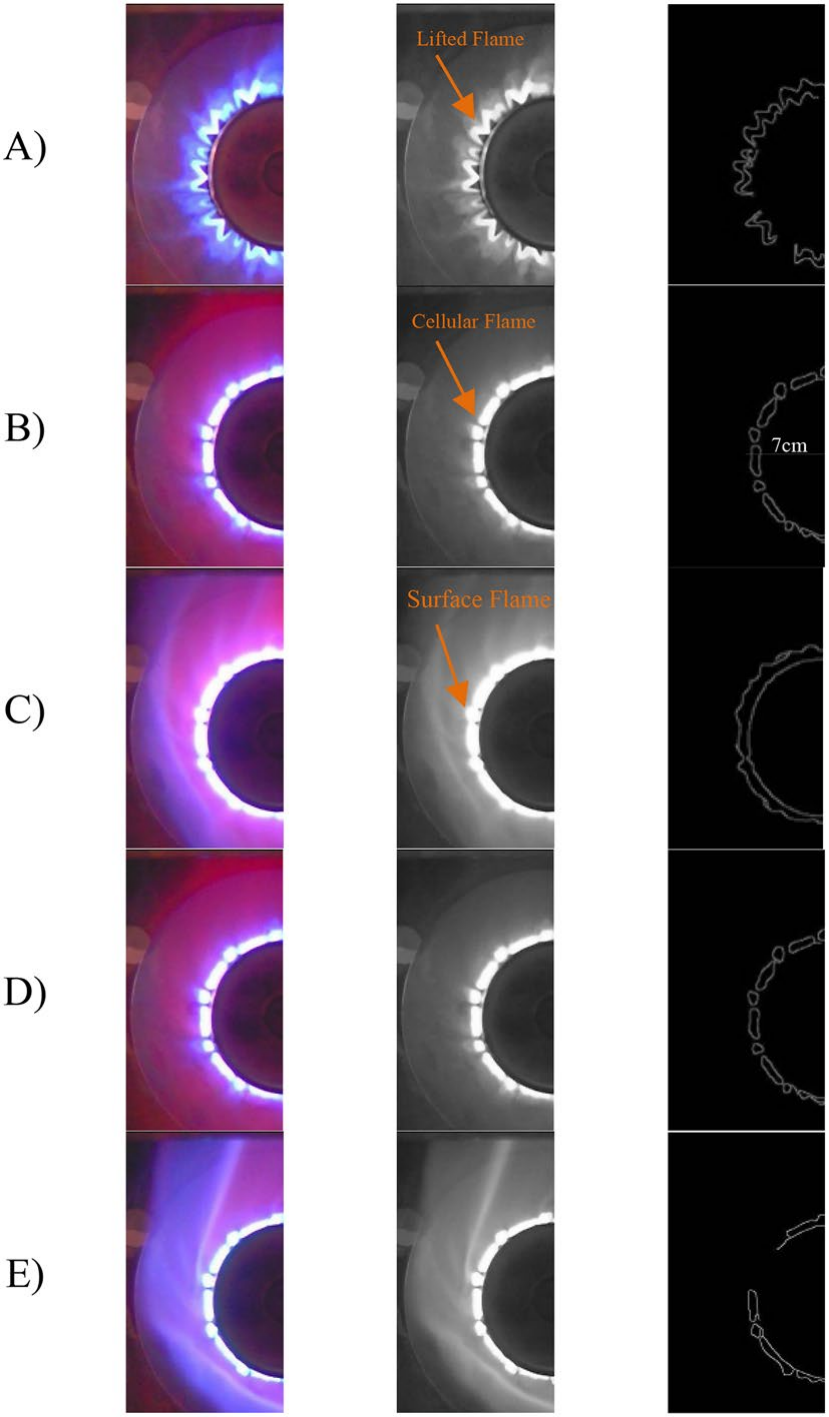
Spectral power diagram of the BPW46 photodiode with an equivalence ratio of 0.79. (**A**) Flame Lift Off; (**B**) Cell Formation; (**C**) Start Surface Flame; (**D**) Re-Formation of Cells; (**E**) The Beginning of Cell Formation.

As shown in Fig. 6, we first have lift-off and turbulence, and then a stable cell flame is formed. In the following equivalence ratio, these cells join together to form the surface flame, in which the steady flame region is located, then the cells reshape, and finally, the flame is lifted off. Zone 1: We have a lift-off, according to the analysis of the torch image from the angle facing it. Zone 2: The cells are shaped and distinguished separately, as seen in flame edge detection from the facing angle. Zone 3: The cells are interconnected and form a uniform surface flame. In this region, OH radiation is maximized and is in the stable region. Zone 4: We observe the re-formation of cells.

Photodiodes operate in a specific wavelength range. Using a photodiode, the intensity of the received light can be obtained in terms of time. Using an amplifier and a data capture system, the current output from the photodiode is converted into voltage, and the time-series signal is digitally recorded at a specific resolution. Changes in the intensity of light received from the flame indicate changes in the concentration of combustion species. An interference filter should be used to express changes in the concentration of a particular species. By measuring the intensity of light received in a wavelength range, especially if the species has chemical light, changes in the concentration of the species can be obtained.

As a natural phenomenon in flame, the maximums created in the frequency diagram are due to the deviation of the flame from the central axis and the change in the frequency of rotation of the input current. Generally, there are limits for different flames in different regimes in which the flame has the maximum frequency. Maximum frequency means the highest amplitude location. This maximum is the maximum or first frequency maximum. At frequencies above the frequency range, there are frequency maxima that are usually due to changes in the speed of the input mixture.

The incomplete combustion of fossil fuels produces carbon monoxide. This contaminant combines with hemoglobin in the blood to produce carboxyhemoglobin. If the number of these molecules in our body increases, it will lead to death. There are different types of nitrogen compounds with oxygen or nitrogen oxides, but the most important ones are in household burners, including $NO2$ and NO. The amount of $NO$ in the blood causes shortness of breath, cyanosis, or death.

In conducting this experiment, two important goals for the research were drawn.

1. Examining cell and surface flame in different equivalence ratios
2. Investigating the level of pollution production

**Figure 7:**
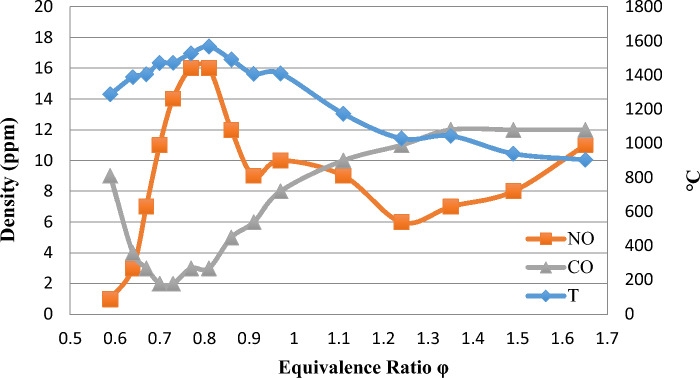
Changes in pollutant concentrations *NO* and *CO* concerning temperature in the ratio of different values at 17.14 kW.

In this research, to achieve the second goal, Testo350 gas analyzer with sensor device was used to obtain the production rate of 1 and 2 and the production process of these two pollutants according to the temperature, which is shown in Fig. 7. By checking the Fig. 7, we found that at the temperature where the burner flame is superficial, the production value of NO has reached the minimum and the production value of CO reaches its maximum value.

Figure 7 shows that a large CO ratio is produced at low equivalence ratios. It is also caused by incomplete combustion. With an increasing homogeneity ratio and complete combustion, the production rate of CO is reduced (CO production decreases with an increasing homogeneity ratio and complete combustion). After passing the stoichiometric equivalence ratio, incomplete combustion increases the CO production process because there is not enough air for combustion reactions in the range of flames above stoichiometric. The minimum production value of CO is in the range of an equivalence ratio of 0.65–0.85. The amount of pollutant CO decreases with increasing power. The burner used in this research produces fewer CO pollutants at a capacity of 17.14 kW compared to lower capacities. This burner releases more heat at higher power while producing fewer pollutants at higher power.

The values of NO and CO depend on temperature. At the equivalence ratio of 0.81, in which we have reported the highest temperature value, the value of NO is at its highest value, i.e., 16 ppm, and the value of CO is almost at its lowest value (2 ppm) (Fig. 7).

The formation of cellular flames causes a sharp decrease in the natural frequency of oscillations. In other words, cellular flames are formed with a drastic reduction in the frequency of oscillations.

**Figure 8:**
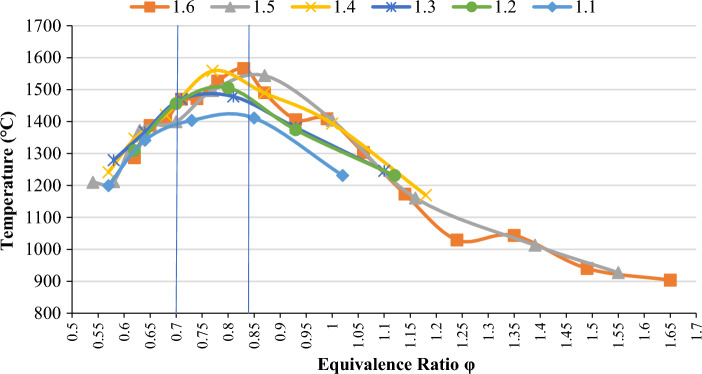
Relationship between temperature and equivalence ratio in different heat capacities.

As shown in Fig. 8, some power is short because we cannot increase the ratio of the gas and air rotameter more than that. Gas and air rotameters have a limited rating; therefore, in some of the powers, the graph is shorter, and in some, it is longer.

**Figure 9:**
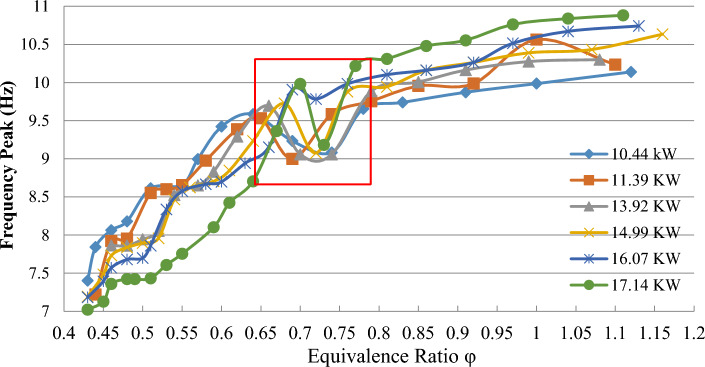
Relationship between natural oscillation frequency and equivalence ratio in different thermal capacities.

In the region where we have a decreasing trend in the frequency peak ratio in the homogeneity ratio (0.7–0.74), which indicates the transition from the cell flame to the surface, the temperature graph increases. In this range, the equivalence ratio in all powers is increasing, and according to the diagram of the stable starting point, which is after the drop point of the frequency peak, it overlaps when the temperature reaches its peak point (Fig. 9).

At low equivalence ratios and in cellular flames, when the flow rate increases, the heat transfer rate to the burner surface decreases because the distance between the burner surface and the premixed flame increases. Heat transfer is decisive in premixed flames at higher equivalence ratios than in surface flames due to the weaker thermal expansion.

The perturbation-temperature effects of a destabilizing factor are low in equivalence ratios and significantly affect the flame instability of methane and air premixes.

## Conclusions

In this study, the sustainability of the surface flame torch was investigated using a photodiode and a data collection system. First, in six different heat capacities and their corresponding equivalence ratios, the temporal distribution of light intensity was obtained by the photodiode and the data collection system, and then, by rapid Fourier transform and intensity transfer from time–space to frequency-space in a specific frequency band, between 7 and 11 Hz, the natural frequency of the oscillations (peak frequency) was measured.

- In a surface flame, as the flow rate (heat capacity) increases, the natural frequency of the oscillations also increases because the velocity of the hot gases burned increases as the flow rate increases. In cell flames, the natural frequency of oscillations decreases with an increasing current rate (heat capacity).
- At the same heat capacities (flow rates), a sharp decrease in the natural frequency of the oscillations indicates the emergence of cellular flames, so we can find the transition from a flat flame to a cellular flame.
- When at a constant heat capacity (flow rate), with an increasing equivalence ratio, we see a decrease in the natural frequency of oscillations, and the transition from cell flame to flat flame occurs. The start of transfer from cell flame to flat flame in thermal capacities of 10.44, 11.39, 12.36, 13.28, 23.23, and 15.18 kW in equivalence ratios of 0.69, 0.69, 7 0, 0.72, 0.72, and 0.73 occurs. The starting point of the transition from the cell flame to the flat flame corresponds to the beginning of the rise zone based on objective observations.
- A range in the equivalence ratio of 0.6–0.8 was specified in the frequency peak. In this range, the cell flame becomes a surface flame.
- In pollutant analysis, the higher the temperature, the lower the CO, and the higher the NO. In the area of flame stability, contaminants are within the allowable range.
- As the flow rate (heat capacity) increases, the range of the cell flame becomes wider since the level of instability increases as the combustion rate increases.
- The inherent instability of premixed flames mainly includes the hydrodynamic effects of thermal expansion between the flame front, and the perturbation-temperature effects of mass overheat penetration (Lewis number less than one).

## Data Availability

The datasets generated and/or analyzed during the current study are not publicly available due to [REASON WHY DATA ARE NOT PUBLIC] but are available from the corresponding author on reasonable request.

## References

[1] Laguillo S, Ochoa JS, Tizné E, Pina A, Ballester J, Ortiz A (2021). CO emissions and temperature analysis from an experimental and numerical study of partially premixed methane flames impinging onto a cooking pot. J. Nat. Gas Sci. Eng..

[2] Wichangarm M, Matthujak A, Sriveerakul T, Sucharitpwatskul S, Phongthanapanich S (2020). Investigation on thermal efficiency of LPG cooking burner using computational fluid dynamics. Energy..

[3] Feng XB, Xu HJ (2020). Modeling the propane combustion process within a micro-catalytic porous combustor by using the lattice Boltzmann method. J. Thermal Anal. Calorimetry..

[4] L. K. Kaushik, P. Muthukumar (2020). Thermal and economic performance assessments of waste cooking oil/kerosene blend operated pressure cook-stove with porous radiant burner. **206**, 118102.

[5] Saberi Moghaddam MH, Saei Moghaddam M, Khorramdel M (2017). Numerical study of geometric parameters affecting temperature and thermal efficiency in a premix multi-hole flat flame burner. Energy..

[6] Lee PH, Lee JY, Han SS, Park CS, Hwang SS (2009). Formation of lean premixed flat flame using cylindrical porous metal plate burner. J. Thermal Sci. Technol..

[7] Kadowaki S, Ohkura N (2008). Time series analysis on the emission of light from methane-air lean premixed flames: Diagnostics of the flame Instability. Trans. Japan Soc. Aeronautical Space Sci..

[8] Cheng TS, Wu CY, Li YH, Chao YC (2006). Chemiluminescent measurements of local equivalence ratio in a partially premixed flame. Combust. Sci. Technol..

[9] Kaewpradap A, Kadowaki S (2007). Heat-loss effects on the chaotic behavior of cellular premixed flames generated by intrinsic instability. J. Thermal Sci. Technol..

[10] Kaewpradap A, Kadowaki S (2010). Instability influenced by CO_2_ and equivalence ratio in oxyhydrogen flames on flat burner. Combust. Sci. Technol..

[11] Kaewpradap A, Jugjai S (2019). Experimental study of flame stability enhancement on lean premixed combustion of synthetic natural gas in Thailand. Int. J. Renew. Energy Res..

[12] Kaewpradap A, Kadowaki S, Nakatsuru S, Nogi R, Katsumi T, Sato D (2021). The shape and fluctuation of hydrogen-propane-butane-air lean premixed flames formed on a flat flame burner. J. Thermal Sci. Technol..

[13] de Paulo JM, Barros JEM, Barbeira PJS (2016). A PLS regression model using flame spectroscopy emission for determination of octane numbers in gasoline. Fuel.

[14] Nomi Y, Gotoda H, Kandani S, Almarcha C (2021). Complex network analysis of the gravity effect on premixed flames propagating in a Hele-Shaw cell. Phys. Rev. E.

[15] Parameswaran T, Hughes R, Gogolek P, Hughes P (2014). Gasification temperature measurement with flame emission spectroscopy. Fuel.

